# Additional Remarks on the Supposed Influence of the Moon in Fevers

**Published:** 1787

**Authors:** Robert Jackson

**Affiliations:** Physician at Stockton


III. *Additional Remarks on the ſuppoſed Influence of the Moon in Fevers. Communicated in a Letter to Dr.* Simmons *by* Robert Jackſon, *M. D. Phyſician at Stockton.*

TO the paper on the influence of the moon in fevers, or rather in anſwer to the letter of Dr. Lind, inſerted in the ſecond part of the the Medical Journal*, allow me, Sir, to add a few remarks. From Dr. Lind's inaugural diſſertation, which I read when but a young man, I muſt acknowledge it was that I firſt received a hint of the moon's influence on the relapſe of the fevers of India; and an opportunity offering to me early in a different part of the world, I was happy to be able, not only to verify what he had taken notice of, but I hope I have had the good fortune to extend the obſervation farther; and the evidence I have produced in ſupport of it, to me at leaſt, ſeems to reſt on no precarious footing.

Dr. Lind, who had the merit of bringing the knowledge of the fact to Europe, ſeems now to have changed his opinion with regard to the cauſe of it; and I cannot help thinking he has changed it without ſufficient reaſon. I would contend with no man about a word; and whether we ſuppoſe the increaſe of fevers, obſerved to happen about the time of new and full moon, owing to the immediate influence of the moon, or to ſome other cauſe connected with it, to me is perfectly indifferent. I would only wiſh it to be known that what I have obſerved in the ſeveral countries in which I have lived, will not

* Page 145.

ſuffer me to allow the cauſe to be merely local, or ſolely owing to the immediate effect of tides overflowing the low grounds. As it is truth I ſeek, not controverſy, I will, indeed, own, that at Savanna la Mar, in Jamaica, which you know is ſituated near the ſea, the connexion of the moon with fevers is more remarkable than in any other part of the world where I have been; yet I muſt at the ſame time add, that the riſe of the tide there ſcarcely ever amounts to eighteen inches. That a tide ſo ſcanty, on a ſandy beach, is likely to produce effects ſo conſiderable, few, I preſume, will be inclined to believe; but leſt any one ſhould, what I can with confidence affirm of the ſame connexion being found, though in a leſs remarkable degree, in the interior parts of America, within a hundred miles of which no tide ever reached, puts the queſtion beyond diſpute.

That the connexion is not local, or confined to the countries within the tropics, not only what I have obſerved in the higher latitudes of America; but to omit others of my own, an obſervation that I have lately met with, in a Treatiſe on the Intermitting Fevers of the Netherlands, by Dr. Grainger, affords the moſt unequivocal proof. Dr. Grainger, deſcribing the the progrefs of the intermitting fever in the year 1748, has the following remark:—"Neque filentio prætereundum, quod die, quo "fol defecit, viginti recens corripiebantur*." This is but a bare fact; but it is a fact of much importance in the prefent queftion. Whoever is curious may look into the book. The ficknefs began on the 9th of July, at what diftance from the full moon will require no great trouble to find out.

It is almoft unneceffary to fay any thing with regard to Dr. Lind's reafoning about the immediate effect of tides. That a high tide leaves behind it what proves a fource of future difeafe I willingly allow; but that the high tide of to-day can be the caufe of fever to-morrow, or even of next day, is fo contrary to experience, that I can by no means affent to it. I have frequently had the opportunity of feeing healthy men brought to unhealthy fituations, and I have conftantly obferved fome time intervene before the appearance of difeafe. As the ftate of the febrile caufe was more or lefs concentrated, or as the body was more or lefs predifpofed, the diftance of time was greater or lefs; but in no one inftance, unlefs perhaps in fome few cafes

* Hiftor. Febris anomalæ Batavæ. p. 21.

of of relapfe, have I ever found the attack of fever inftantaneous.

*Stockton,*
July 31, 1787.

